# The Effect of Dihydromyricetin (DMY) on the Mechanism of Soy Protein Isolate/Inulin/Dihydromyricetin Interaction: Structural, Interfacial, and Functional Properties

**DOI:** 10.3390/foods13213488

**Published:** 2024-10-30

**Authors:** Puyu Chen, Hairong Bao

**Affiliations:** College of Food Science & Technology, Shanghai Ocean University, Shanghai 201306, China; cpyfighting@163.com

**Keywords:** soybean protein isolate, inulin, dihydromyricetin, ternary complexes, structure, emulsifier, antioxidant properties

## Abstract

The combination of proteins with polysaccharides and polyphenols is expected to improve their physicochemical and functional properties. In this study, a novel plant-based antioxidant emulsifier was formed by soybean protein isolate (SPI), inulin (INU), and dihydromyricetin (DMY). Based on the binary system of SPI/INU, we focused on exploring the effect of the DMY concentration (0.5 mg/mL~2.5 mg/mL) on the formation and properties of the ternary complex. The structure, interaction mechanism, and interfacial and functional properties of the ternary complex were investigated. The results indicate that compared to the SPI/INU binary complex, the SPI/INU/DMY ternary complex had a significant decrease in particle size (~100 nm) and a slight decrease in absolute zeta potential. The SPI/INU binary complex with DMY mainly interacted by hydrogen bonding and hydrophobic interactions. Due to the incorporation of DMY, the structure of SI was denser and more flexible. The ternary complex exhibited an ideal three-phase contact angle and demonstrated better foaming and antioxidant ability. Additionally, compared to SPI/INU, the ternary complex had a significant improvement in EAI. These results provide a strategy for polyphenols to modify the structure, interfacial properties, and functions of protein/polysaccharide complexes. This provides a potential reference for the preparation of more ternary complexes with excellent emulsifying and antioxidant properties for application in emulsions.

## 1. Introduction

Food-grade colloidal-particle-stabilized Pickering emulsions are increasingly being used to modify texture, reduce fat, and deliver beneficial compounds. This is because of their prolonged stability, inexpensiveness, and ability to firmly attach to the oil–water interface [1]. Moreover, with the increasing consumer demand for clean labels, using natural materials and biopolymers instead of surfactants to stabilize emulsions is the current trend. It has been found that protein, polysaccharide, and polyphenol biomolecules are beneficial to health and can stabilize Pickering emulsions. However, some characteristics of individual biomolecules make their use in Pickering emulsions challenging, such as the inherent sensitivity of proteins and the lack of interfacial activity of polysaccharides [2]. An effective way to overcome this problem is to construct multi-component particles (two or more compounds of a complementary nature). Recently, protein/polysaccharide/polyphenol ternary complex research has received increasing attention due to its better functional and structural properties [3]. For example, the soy protein isolate/carrageenan/quercetin ternary complex is better at stabilizing the emulsion and reducing the oxidative degradation of beta-carotene than the soy protein isolate/carrageenan binary complex [4]. When using soy isolate protein/citrus pectin/gallic acid ternary complexes to stabilize emulsions, it was discovered that the ternary complexes can enhance the properties of the emulsion more than binary complexes [5]. The addition of polyphenols can strengthen the connections between proteins and polysaccharides, as well as enhance the antioxidant capabilities of protein-based complexes. A recent research trend has been the introduction of ternary complexes consisting of more types of proteins, polysaccharides, and polyphenols [3].

Plant proteins are becoming increasingly favored as a substitute for animal proteins due to their greater sustainability and lower environmental effects. Soy protein isolate (SPI) is a popular plant protein in food, agriculture, and biotechnology due to its rich nutritional content, inexpensive production, and biocompatible nature [6]. However, compared to animal proteins (casein, whey proteins, etc.), SPI’s stabilizing properties in emulsions are poor. SPI solutions have a short storage time, with aggregation and oxidation of the solution under the influence of temperature and oxygen [7]. Modifying or disrupting the structure of SPI’s basic globular proteins (soybean globulin and β-associated soybean globulin) is crucial for increasing its emulsifying properties and stability. Several studies found that the Maillard reaction between proteins and reducing sugars may benefit the functional characteristics and stability of proteins [8]. Furthermore, protein–polyphenol interactions are a potential way to promote the emulsification and antioxidant capacity of proteins [9].

Inulin (INU) is a native multifunctional polysaccharide (prebiotic) found in many plants that is simple to access, inexpensive, and known to have multiple physiological functions. Adequate intake of INU can reduce the risk of intestinal diseases and arteriosclerosis, and it also promotes the absorption of iron, magnesium, and calcium [10]. It has been shown that interactions with INU promote the gelling properties and emulsification of proteins [11]. The addition of inulin to β-globulin improved the surface activity of proteins [12]. However, complexes’ stability is influenced by particle size and intermolecular interactions. Exposing the protein to more binding sites can be beneficial. This greatly alters the functional features of the protein and allows it to bind tightly to the polysaccharide, thus enhancing stability [13]. Currently, the treatment of proteins includes physical, chemical, biological, and genetic methods that can help to expose more protein binding sites. After treatment with ultrasound, for example, SPI exposes more binding sites and forms a binary complex with INU that has a lower particle size and higher stability [14]. The pH modification of proteins has been applied due to its efficiency, cost-effectiveness, and ease of operation [15]. Studies have shown that when proteins are treated at different pHs (2, 4, 10, and 12) with ultrasound, pH 12 with ultrasound can reduce the protein size and raise stability. This is because, at pH 12, the protein is further from the isoelectric point and has a large electrostatic repulsion force. It helps more unfoldings of protein structures to form [16]. However, ultrasound tends to expose proteins to extreme temperatures and is not easily commercialized [17]. Conventional heat treatment is a common protein treatment, and an appropriate temperature can cause proteins to dissociate into small aggregates, thus improving stability. Nevertheless, when treated with excessively high temperatures, proteins will undergo large-scale aggregation and precipitation. Treating SPI at 85 °C for 30 min can effectively expose the hydrophobic groups and reduce the particle size, while not causing aggregation [18]. However, there are few studies on the preparation of SPI/INU by combining SPI (after alkali–heat treatment) with INU.

Dihydromyricetin (DMY) is a type of flavonoid and the major bioactive component of *Ampelopsis grossedentata* leaves. It was approved in China in 2013 as a new-resource food with a variety of beneficial functions [19]. A single DMY can stabilize Pickering emulsion gel [19]. Its ability to stabilize emulsions has been significantly improved by combining it with high-straight-chain starch and beet pectin [20,21]. However, the interactions among the types of soy protein isolates, inulin, and dihydromyricetin have only been investigated in binary studies, such as soy isolate protein/inulin, protein/dihydromyricetin, and dihydromyricetin/polysaccharide, and all of them were found to have improved physicochemical and functional properties. Therefore, it is essential to investigate the possibility of combining DMY with polysaccharide/polyphenol systems.

In this study, protein/polysaccharide/polyphenol-based ternary complexes were constructed. Different concentrations of DMY were added to the SPI/INU binary complexes, which were formed by SPI interaction with INU through the Maillard reaction. The impact of the DMY concentration on the physicochemical, structural, interfacial, and functional properties of the complexes was investigated. The turbidity, particle size, and zeta potential of the complexes were analyzed. The structure was investigated using FTIR spectroscopy, ultraviolet absorption spectroscopy, endogenous fluorescence spectroscopy, and SDS-PAGE. The interfacial properties of the ternary complexes were analyzed, and their functional properties were investigated using the DPPH, ABTS, and OH free radical scavenging rates and differential scanning calorimetry (DSC). This study potentially constructs a new and effective soybean protein isolate/inulin/dihydromyricetin (SID) antioxidant emulsifier. It provides valuable insights into a strategy for polyphenols to modify the structure, interfacial properties, and functions of protein/polysaccharide complexes. It also serves as a reference for building protein/polysaccharide/polyphenol-based emulsifiers.

## 2. Materials and Methods

### 2.1. Materials

Soybean protein isolate (food grade, SPI, >90% protein content) was purchased from Jiahe Xu Ri Foods Co., Ltd. (Shandong, China), inulin (food grade, INU, ≥99%) from COSUCRA Groupe Warcoing SA (Warcoing, Belgium), dihydromyricetin (food grade, DMY, 98%) from Shanxi Yuzhou Bio-technology Co., Ltd. (Shanxi, China), and all other chemicals were analytical grade.

### 2.2. Preparation of SID Ternary Complexes

#### 2.2.1. Preparation of Soybean Protein Isolate (SPI) Dispersion

The SPI solution was prepared following a previous method with slight modifications [22]. First, 4 g of SPI was mixed with 200 mL of deionized water (20 mg/mL) and agitated at 300 rpm for 1 h at 25 °C using a digital thermostatic magnetic stirrer (HMS-203D, Shanghai Huxi Instrument Co., Ltd., Shanghai, China), which ensured complete dissolution of the protein. The pH of the solution was set to 12.0 by adding 1 M NaOH, then agitated at 300 rpm for 1 h. The resulting dispersion solution was heated to 85 °C and agitated for 30 min (300 rpm) to promote further hydration of the proteins, followed by cooling to room temperature. The solution’s pH was then set to 7.0 (1 M HCl) and refrigerated overnight at 4 °C before use.

#### 2.2.2. Preparation of Binary Complex of Soybean Protein Isolate/Inulin (SI)

The binary complexes followed Li et al.’s method with slight modifications [3]. First, 4 g of inulin (INU) was mixed with 200 mL of deionized water (20 mg/mL) and agitated at 300 rpm for 30 min at 25 °C to create a homogeneous solution. The pH was then adjusted to 7. Then, the SPI and INU solutions were combined in a 1:1 (*v*/*v*) ratio and agitated at 300 rpm for 2 h at 70 °C before being promptly cooled to stop the reaction.

#### 2.2.3. Preparation of a Ternary Complex of Soy Protein Isolate/Inulin/Dihydromyricetin (SID)

**Figure 1:**
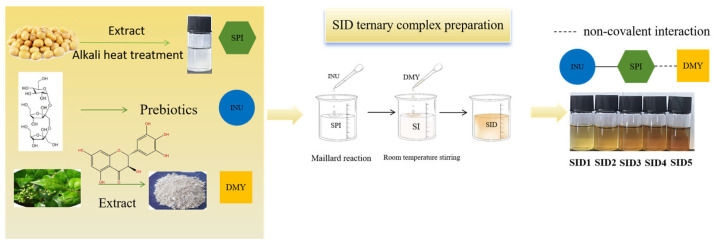
Preparing ternary complexes of soy protein isolate/inulin/dihydromyricetin (SID).

DMY solutions were prepared by dissolving DMY in 100 mL of deionized water to produce polyphenol solutions with concentrations of 1 mg/mL to 5 mg/mL, stirring at 80 °C for 1 h at 300 rpm, and adjusting the pH to 7. Equal volumes were added to the dispersion of soybean protein isolate/inulin (SI) and agitated for 3 h at 25 °C. The obtained suspensions were freeze-dried in a vacuum at −50 °C for 48 h (CHRIST ALPHA1-2 Vacuum Freeze-Dryer, Marin Christ, Osterode am Harz, Germany). Finally, the ternary complex solutions were formed with a dihydromyricetin concentration of 0.5 mg/mL to 2.5 mg/mL (marked as SID1~SID5). Figure 1 shows the preparation of the complex.

### 2.3. Physicochemical Properties of Complexes

#### 2.3.1. Turbidity Measurement

The turbidity of the solutions was evaluated at 600 nm through a UV-Vis spectrophotometer (T6 New Century UV Spectrophotometer, Beijing Pulse General Instrument Co., Ltd., Beijing, China).

#### 2.3.2. Particle Size and Zeta Potential

The particle size and zeta potential were determined using a laser particle sizer (Malvern Zetasizer Pro, Malvern, Vienna, Austria).

### 2.4. Structural Properties of Complexes

#### 2.4.1. Fourier Transform Infrared Spectroscopy (FTIR)

The infrared spectra of samples were examined using FTIR (Nicoletis-50 FTIR spectrometer, Thermo Fisher Scientific, Shanghai, China) at 500 cm^−1^–4000 cm^−1^. The PeakFit software v4.12 was utilized to evaluate the spectra, to calculate the changes in the samples’ secondary structure [23].

#### 2.4.2. UV Absorption Spectroscopy Detection

Using 0.1 M phosphate buffer, the protein concentration of the samples was set to 0.25 mg/mL. A UV spectrophotometer (Shimadzu UV2600, Shimadzu Corporation, Kyoto, Japan) was used to detect UV absorption spectra from 200 to 500 nm at room temperature [3].

#### 2.4.3. Endogenous Fluorescence Spectroscopy Detection

Using 0.1 M phosphate buffer, the protein concentration of the samples was set to 0.25 mg/mL. The fluorescence spectrum was detected using three-dimensional fluorescence spectroscopy (Hitachi F-7100, Hitachi High-Technologies Corporation, Tokyo, Japan) with an emission wavelength of 295 nm, an excitation wavelength range of 300–500 nm, and a slit width of 5 nm.

#### 2.4.4. SDS-PAGE Determination

Protein and complex solutions with a protein content of 5 mg/mL were prepared, and 10 μL of sample solution was blended with the upload buffer at a volume ratio of 1:1 and boiled for 5 min. The upload volume was 10 μL, and the concentrations of separating gels and concentrated gel were 12% and 3%, respectively. Electrophoresis was applied at 120 V for 1–2 h. Staining was carried out with Kaomas Brilliant Blue R-250 (Affinibody LifeScience AG, Wuhan, China) for 4 h [24].

#### 2.4.5. Scanning Electron Microscope (SEM)

The microstructures of the samples were observed via an SEM (Hitachi SU5000, Hitachi High-Technologies Corporation, Japan) at an operating voltage of 15.00 kV [23].

### 2.5. Interfacial Properties of Complexes

#### 2.5.1. The Three-Phase Contact Angle

A 1% solution was uniformly distributed on a smooth and flat glass plate and dried in a blast drying oven (Shanghai Huitai Instrument Manufacturing Co., Ltd., Shanghai, China) at 50 °C for 1 h. Then, the homogeneous films were created. The contact angle of water droplets in the ambient atmosphere with the complex film was determined using an SDC-100 device (Dongguan Ding Sheng Precision Instrument Co., Ltd., Dongguan, China) following the method described by Wu et al. [25] with a few changes.

#### 2.5.2. Emulsifying Property

The sample solution was combined with soybean oil in a 1:1 (*v*/*v*) ratio via a high-shear homogenizer (IKA T10 disperser, IKA/Ayer, Staufen, Germany) at 10,000 rpm for 1 min. After dispersing 100 μL of the emulsion in 10 mL of 0.1% (*w*/*v*) SDS solution, absorbance was measured at 500 nm [7]. The emulsifying activity index (EAI) and emulsion stability index (ESI) were obtained as follows:

(1) $$\mathrm{EAI}\left(m^{2}/g\right)=\left(2\times 2.303\times \frac{A0\times N}{c\times P\times 10,000}\right)$$

(2) $$\mathrm{ESI}\left(\%\right)=\frac{A0}{A0-A10}\times 10$$

where c is the concentration of the complex, N is the number of dilutions, and P is the percentage of the oil phase that is 0.5; A0 and A10 are the absorbance at 0 min and 10 min, respectively.

#### 2.5.3. Foaming Property

The test solution (10 mL) was mechanically sheared (10,000 rpm, 90 s) using a high-shear homogenizer to generate foam [26]. Foamability and foam stability were obtained for each test sample using the following:

(3) $$\mathrm{Foamability}\left(\%\right)=\frac{V0}{VT}\times 100$$

(4) $$\mathrm{Foam}\;\mathrm{stability}(\%)=\frac{V60}{V0}\times 100$$

where VT is the volume of the test sample produced before shearing (10 mL), V0 is the volume of foam at 0 min (mL), and V60 is the volume of foam at 60 min of standing (mL).

### 2.6. Functional Properties of Complexes

#### 2.6.1. Antioxidant Capacity Analysis

DPPH free radical scavenging rate. Referring to Jing et al. [27], 2 mL of complex solution was combined with 2 mL of 0.1 mmol/L DPPH alcohol for 30 min at 25 °C in darkness. The absorbance value of the solution was examined at 517 nm. A sample blank was made by substituting the DPPH solution with ethanol; ethanol was used as a control instead of the tested solution.

(5) $$\mathrm{DPPH}\;\mathrm{free}\;\mathrm{radical}\;\mathrm{scavenging}\;\mathrm{rate}\;\left(\%\right)=1-[\frac{\left(A1-A2\right)}{A0}]\times 100$$

where A1, A2, and A0 are the absorbance of the sample, sample blank, and control, respectively.

ABTS free radical scavenging rate. The ABTS solution (7 mmol/L) was combined with an equivalent volume of K_2_S_2_O_8_ solution (4.9 mmol/L) and kept in the dark at room temperature for 12–15 h. The ABTS solution’s absorbance was adjusted to 0.70 ± 0.02 at 734 nm using deionized water. Then, 3 mL of the ABTS solution was combined with 50 μL of sample solution. After 6 min of treatment, the mixture’s absorbance was measured at 734 nm. The ABTS scavenging activity (%) was calculated using the following equation:

(6) $$\mathrm{ABTS}\;\mathrm{free}\;\mathrm{radical}\;\mathrm{scavenging}\;\mathrm{rate}\;\left(\%\right)=1-[\frac{(A1-A2)}{A0}]\times 100$$

where A1, A2, and A0 are the absorbance of the sample, sample blank, and control, respectively.

OH free radical scavenging rate. The sample solution was combined with 1 mL of phenanthroline (1.5 mmol/L) and 2 mL of PBS (20 mmol/L, pH 7.4). Then, 1 mL of ferrous sulfate (0.5 mmol/L) and 1 mL of hydrogen peroxide solution (0.1% *v*/*v*) were added. The combination was incubated at 37 °C for 50 min, and the mixture’s absorbance was measured at 536 nm. The OH free radical scavenging rate of hydroxyl radicals was calculated as follows:

(7) $$\mathrm{The}\;\mathrm{OH}\mathrm{free}\;\mathrm{radical}\;\mathrm{scavenging}\;\mathrm{rate}\;\left(\%\right)=\frac{A1-A0}{A2-A0}\times 100$$

where A1, A2, and A0 are the absorbance of the sample, sample blank (without hydrogen peroxide solution), and control (without sample solution), respectively.

#### 2.6.2. Thermal Stability

A differential scanning calorimeter (DSC8500, PerkinElmer, Waltham, MA, USA) was used to detect the thermal properties of the samples. Lyophilized samples (1–4 mg) were weighed and sealed in aluminum pots for measurement. Each pot was heated from 40 °C to 200 °C under a nitrogen atmosphere at 10 °C/min [27].

### 2.7. Statistical Analysis

All data measurements were repeated three times per sample and analyzed with SPSS 13.0 software. Significant differences among the means were analyzed by one-way ANOVA and Tukey’s test, with a significance level of *p* < 0.05. GraphPad Prism 9.5.0 software was used for graphical processing.

## 3. Results and Discussion

### 3.1. Physicochemical Properties of SPI-INU-DMY Complexes

#### 3.1.1. Turbidity

**Figure 2:**
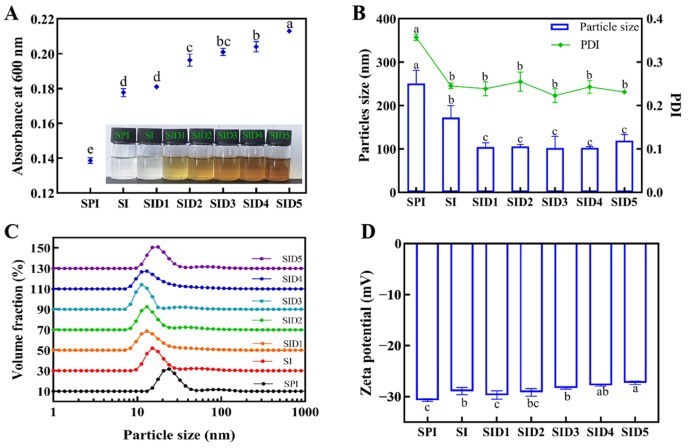
Turbidity (**A**), particle size, PDI (**B**), volume distribution (**C**), and zeta potential (**D**) of SPI, SPI-INU (SI) binary complex, and SPI-INU-DMY (SID) ternary complexes at different DMY concentrations. Different lowercase letters (a–e) indicate significant differences (*p* < 0.05).

The sample’s turbidity was assessed using UV–visible spectroscopy, which is responsive to the formation of the complex [26]. The formation of complex colloidal particles results in light wave scattering, thus increasing the turbidity of the liquid. The amount, particle size, and refractive index contrast of the particles determine the degree of the turbidity rise. Figure 2A depicts the sample’s turbidity at different DMY concentrations. The turbidity value increased due to SPI and INU forming soluble complexes [14]. The turbidity of the solution increased as DMY concentrations rose, which was due to the hydroxyl groups of DMY that can bind to protein residues. This helps to unfold the protein’s spatial structure and then promotes the combination of SPI and IUN [28]. The complex suspension shows a uniform light-brown system, and after 78 h of storage, no clear precipitation was observed, and the liquid was still in a uniform colloidal state. This suggests that SID particles have excellent resistance to gravitational forces. When polyphenols bind to protein/polysaccharide complexes, the polyphenols are embedded in the protein/polysaccharide gaps. This helps to construct a denser structure of the protein/polysaccharide complexes, preventing the formation of bigger aggregates due to spatial blockage [29]. This indicates that when polyphenols are added to the polysaccharide/protein system, they mainly act on the protein to change the protein conformation or generate steric hindrance. Similarly, the phenomenon was also observed in the preparation of pea protein/grape seed anthocyanin (PP/GSA) complexes. The turbidity of the sample solution increased with increasing concentrations of grape seed anthocyanin (0–0.3%), and there was no deposition, indicating the formation of colloidal particles with resistance to gravitation [30].

#### 3.1.2. Particle Size and Distribution

Changes in particle size may affect the complex’s specific surface area, surface energy, and exposure of internal groups, hence affecting its structure and characteristics [31]. In general, complex particles should be at least one order of magnitude smaller than the emulsion size. This improves particle adhesion at the oil–water interface, encapsulates oil droplets, and enhances emulsion stability [32]. In Figure 2B,C, the incorporation of DMY significantly reduced the particle size of the complex. All ternary composite particles had a particle size of around 100 nm. This shows that DMY changed SPI’s ability to form colloidal particles by self-assembly to form a compact complex [30,33]. DMY can react with SPI/INU through hydrogen bonding and hydrophobic interactions, embedding into the polysaccharide interstitials or the interstitials between SPI and INU, making the structure more compact [34]. When the DMY concentration was 2.5 mg/mL, the particle size showed a slight increase (118.9 nm). This is because excess DMY becomes the bridging agent for SPI or SI complexes and behaves as polydentate ligands. Thus, it causes proteins to gradually become dimers or polymers [28]. Some more free DMY binds to protein/polysaccharide surfaces and causes intermolecular aggregation of the complexes [35]. This is in agreement with the results of the influence of the epigallocatechin gallate (EGCG) concentration on the particle size of seabass proteins [36]. When combining apple polyphenols with SPI, the same trend was observed as the concentration of apple polyphenols increased [7].

#### 3.1.3. Zeta Potential

An indicator to assess a complex’s stability is its zeta potential [12]. The complexes in this study all have a negative potential (Figure 2D) because SPI carries a negative charge under neutral conditions, and DMY also carries a negative charge (−22.93 ± 2.41 mV). When INU was added, the absolute value of the zeta potential decreased. During the interaction with SPI, INU may shield the negative charge on the protein surface, which reduces the zeta potential of the protein solution [14]. The addition of DMY alters SPI’s ability to self-assemble and reduces the overall amount of net negative charge on the complex’s surface [37]. This could also be due to DMY having a lower net charge than proteins [3]. A zeta potential of close to or less than −30 mV often provides sufficient electrostatic repulsive force to stabilize the emulsion [38]. When DMY was 2.5 mg/mL, the zeta potential of the complex was −27.30 mV. Despite the decrease in charge, it remains close to 30 mV, so SID complexes still have the potential to stabilize the emulsion. Similarly, when proanthocyanidins were added to the whey isolate, the protein/dextran system experienced a net charge reduction [3].

### 3.2. Structural Properties of SPI-INU-DMY Complexes

#### 3.2.1. FTIR

**Figure 3:**
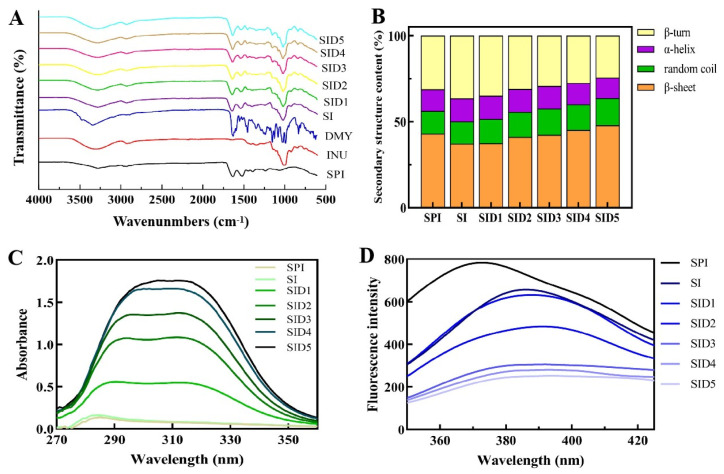
FTIR spectra (**A**), secondary structure (**B**), ultraviolet absorption spectra (**C**), and fluorescence spectra (**D**) of SPI, SPI-INU (SI) binary complex, and SPI-INU-DMY (SID) ternary complexes at different DMY concentrations.

FTIR can identify alterations in functional moieties, hence facilitating the analysis of complex binding. The FTIR spectra of all samples are shown in Figure 3A. It was clear that SPI’s FTIR spectrum had three main bands: the amide I band at 1631.79 cm^−1^ (hydrogen bonding and C=O stretching), the amide II band at 1517.66 cm^−1^ (N-H bending), and the amide A band at 3274.79 cm^−1^ (hydrogen bonding and C-H stretching). The main absorption peak of DMY is at 3336.62 cm^⁻1^. This is because of the stretching vibration of -OH in the polyphenol structure [3]. The hydrogen bonding vibration is usually seen at 3100~3400 cm^−1^. Compared to SPI, the amide A band of the SI complex shifted from 3274.79 cm^−1^ to 3277.13 cm^−1^. This shows that hydrogen bonds exist between the amide group in SPI and the carboxyl or hydroxyl in INU [1]. The sharp peak of DMY turned into a smooth peak after it was added to the SI complex, indicating the presence of non-covalent hydrophilic/hydrophobic interaction forces in the complex [39]. In the SID complex, the amide A band was shifted to different extents, further suggesting the involvement of hydrogen bonding among the complexes. In addition, the amide I and amide II bands of the protein were shifted from 1631.79 cm^−1^ to 1636.55 cm^−1^ and from 1517.66 cm^−1^ to 1536.68 cm^−1^, respectively, and accompanied by a decrease in peak intensities. This change mainly reflected the hydrophobic interactions between the complexes [22].

Alterations in the protein’s secondary structure may affect its amide I and amide II regions. In comparison to the amide II band, the amide I band is more sensitive to changes in the protein’s secondary structure [40]. The secondary structure is represented by the following individual subpeaks: 1615–1637 cm^−1^ is β-sheet, 1664–1681 cm^−1^ is β-turn, 1646–1664 cm^−1^ is α-helix, and 1637–1645 cm^−1^ is random coil [41]. As shown in Figure 3B, the addition of DMY resulted in decreases of 10.18% and 21.78% for the α-helix and β-turn, respectively, and increases of 11.33% and 20.20% for the β-sheet and random coil. An increase in β-sheet content may contribute to SID complexes’ hemi-mobility and flexibility, which will improve their rheology, foaming qualities, and emulsification [36]. SPI’s conformation changed from α-helix to random coil, resulting in a more open and looser structure [7]. It shows that the addition of DMY has an impact on the secondary structure of SI. Similarly, when tannic acid is added to lactoferrin, there is a reduction in the α-helix content of protein, probably because of the binding of hydrophobic areas [42].

#### 3.2.2. Ultraviolet Absorption Spectroscopy

UV spectroscopy can determine changes in the tertiary structure of proteins. By observing the area size of the UV absorption peaks, the relationship between proteins and their interactions with small molecules can be analyzed. The changes in the maximum peaks in the UV absorption spectra may be due to changes in protein skeleton conformation and α-helix content, thus altering the microenvironment of aromatic amino acid residues [43], possibly because when polyphenols interact with proteins, it leads to protein unfolding. This will impact the distribution of Tyr and Trp (internal non-polar) in proteins. It may also be because the amide group of the protein interacts with the phenolic hydroxyl group of polyphenols, leading to a change in hydrogen bonding [44]. Try, Tyr, Phe, and sulfur-containing amino acids showed absorption peaks in the 230–310 nm wavelength area [45]. Figure 3C shows the UV absorption spectra of the sample. The ternary complexes have absorption peaks at around 320 nm for the conjugated double bond, carbonyl aromatic ring structure, or biphenyl-type structure, probably because the light at 320 nm is absorbed by structures such as the C=O bond and carbonyl group in DMY. When DMY was added, the UV absorption spectrum shifted and increased. This shows the exposure of protein tyrosine and tryptophan residues and a looser protein structure [46]. This red shift in the UV spectrum is due to protein unfolding and interaction with DMY by hydrogen bonds [47]. This suggests that Tyr and Trp residues were exposed and the polarity of the surrounding microenvironment changed [7]. Protein emulsification is related to the distribution of hydrophobic groups. As the protein unfolds, its hydrophobic groups are exposed. This can enhance its surface activity and enable it to better adsorb onto the oil droplet surface, resulting in an increased protein emulsifying activity index (EAI) [48].

#### 3.2.3. Endogenous Fluorescence Spectroscopy

The tertiary structure changes of proteins are often characterized through fluorescence spectroscopy [49]. Aromatic amino acid residues are responsible for protein fluorescence. As the ligand connects to the protein and affects the emission of intrinsic fluorescent groups, fluorescence quenching occurs [50]. Figure 3D shows the fluorescence spectra of the samples. SPI has a maximum emission peak of around 372 nm. A decrease in fluorescence intensity occurs with the addition of INU because the fluorescence signal of tryptophan residues is blocked by the shielding effect of sugar [51]. Similarly, the fluorescence intensity of pea protein decreased after adding maltodextrin [52]. The fluorescence intensity of the SID ternary complex decreased compared to the SI complex. This is because of the hydrophobic interaction between polyphenols and tryptophan residues of proteins and the shielding of hydrophobic groups caused by protein unfolding [53]. The unfolding of the protein structure can enable its hydrophobic groups to be immersed in the oil phase and its hydrophilic groups to be immersed in the water phase to build a liquid film, thereby stabilizing emulsions and improving the EAI [54]. As the protein structure expands and hydrophobic side chains are exposed, surface activity and protein–membrane surface tension will be improved, thus increasing the foaming properties of proteins [49]. With the addition of DMY, the maximum fluorescence emission peak was red-shifted. This result was due to the conformational change of proteins, which exposed more tryptophan (a hydrophobic amino acid) to the polar environment [1]. In a polar environment, the excited-state energy of the chromophore will decrease. The decrease in excited-state energy will lead to a shift of the fluorescence emission wavelength to a longer wavelength direction (red shift). Similar results occurred when different polyphenols were combined with whey proteins [55].

#### 3.2.4. SDS-PAGE

**Figure 4:**
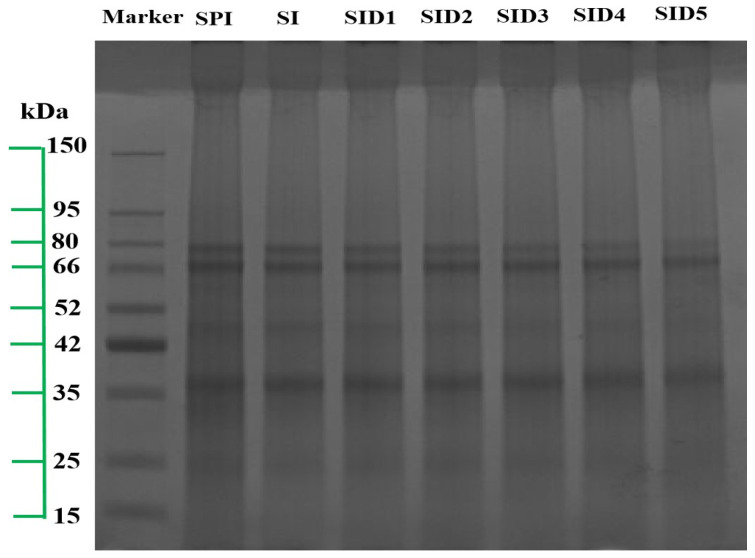
SDS-PAGE electrophoresis of SPI, SPI-INU (SI) binary complex, and SPI-INU-DMY (SID) ternary complexes at different DMY concentrations.

SPI is a mixture of proteins that may be divided by centrifugal force into 2S, 7S, 11S, and 15S fractions [41]. Figure 4 shows the SDS-PAGE gel of all samples. After adding INU, the protein bands did not show obvious changes. However, the bands appeared lighter, indicating that the subunit participated in the Maillard reaction between SPI and inulin [56]. The addition of DMY did not create any new bands, which means that there is no covalent reaction between DMY with SPI/INU [57]. The results are similar to non-covalently linked SPI/anthocyanin complexes [41]. The bands of the coconut globulin/coffee polyphenol (CG-CP) complex are the same as those of CG, and there are no obvious new bands. This suggests that the CG-CP complex is formed by non-covalent interactions [58]. There is a slight fading of the SID4 and SID5 bands, especially for SID5. This may be because high-concentration DMY causes protein aggregation and forms partially insoluble aggregates [59].

#### 3.2.5. Scanning Electron Microscopy (SEM)

**Figure 5:**
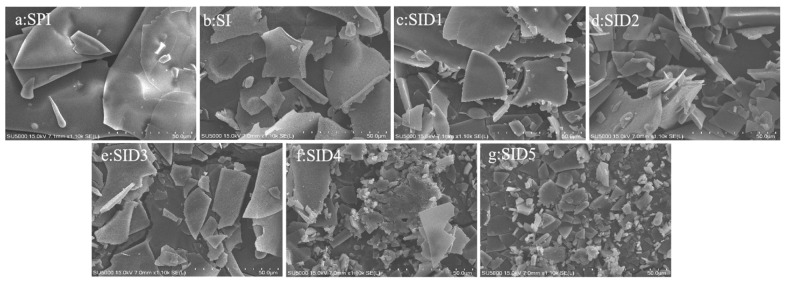
SEM images of SPI, SPI-INU (SI) binary complex, and SPI-INU-DMY (SID) ternary complexes at different DMY concentrations.

SEM was used for observing the microstructure of all samples, as can be seen in Figure 5. SPI had a larger lamellar structure. Adding INU ruined the big sheet construction of SPI. The SID complexes are smaller fragments with uniform and compact structures [55]. When DMY was added, it exposed the protein’s internal hydrophobic groups. This reduced the aggregation of the complexes. When hydrophobic groups were exposed and interacted, they restricted the movement of molecules to aggregate, thus maintaining a stable structure [60]. Similarly, when EGCG formed a ternary complex with soybean isolate protein/soybean polysaccharide, the ternary complex had a smaller lamellar structure [61].

### 3.3. Interfacial Properties of SPI-INU-DMY Complexes

#### 3.3.1. Interfacial Wettability

**Figure 6:**
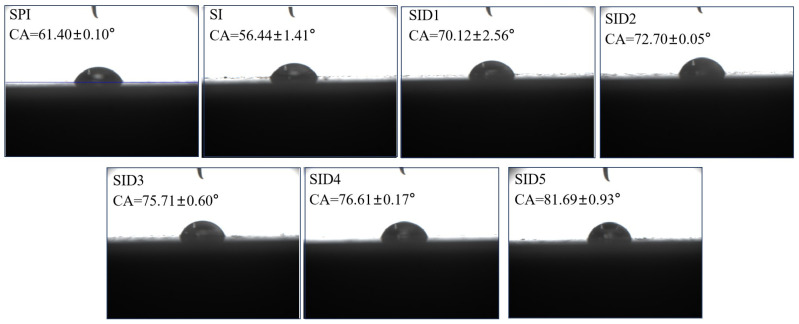
Three-phase contact angles (θ) of SPI, SPI-INU (SI) binary complex, and SPI-INU-DMY (SID) ternary complexes at different DMY concentrations.

In the food industry, Pickering emulsions can be used in applications such as loading and delivering bioactive substances, food packaging, and fat substitutes. However, during the processes of production, storage, transportation, and application, emulsions are likely to encounter diverse instability issues, including stratification, creaming, flocculation, and aggregation. These problems will affect the product’s storage life and sensory properties. The capacity of the particles to stabilize the emulsion depends largely on their wettability. The three-phase contact angle (θ) is frequently applied to determine wettability. Particles with θ > 90° were classified as lipophilic, whereas those with θ < 90° were classified as hydrophilic. Particles with a three-phase contact angle near 90° have an excellent performance in stabilizing the water–oil interface due to better amphiphilicity [23]. As shown in Figure 6, the three-phase contact angle of the SID complexes increased compared to SPI and SI. At 2.5 mg/mL DMY, the three-phase contact angle reached 81.69°. When DMY was added to the SI complex, the complex contact angle slowly approached 90°. This shows that complexes may better adhere at the oil–water interface to stabilize emulsions. DMY unfolded the SPI structure, exposing the interior hydrophobic groups and increasing hydrophobicity [58]. Also, the literature shows that the contact angle of DMY is 118.8°, suggesting that DMY exhibits hydrophobicity [20]. In contrast, the three-phase contact angle of SPI is 61.40°, which is hydrophilic. Thus, the wettability of the particles can be altered by adjusting the DMY concentration. When epigallocatechin-3-gallate (EGCG) was added to sea bass protein, the contact angle of the complex finally reached 78.7°, showing a better performance for the stabilization of the emulsion [36]. The contact angle of the soy protein/β-glucan/coumarin complex changed to 92.6° when coumarin was added, which shows an improvement in emulsifying properties [1]. DMY can improve the wettability of the SI complex and thus enhance its emulsifying properties.

#### 3.3.2. Emulsifying Properties

**Figure 7:**
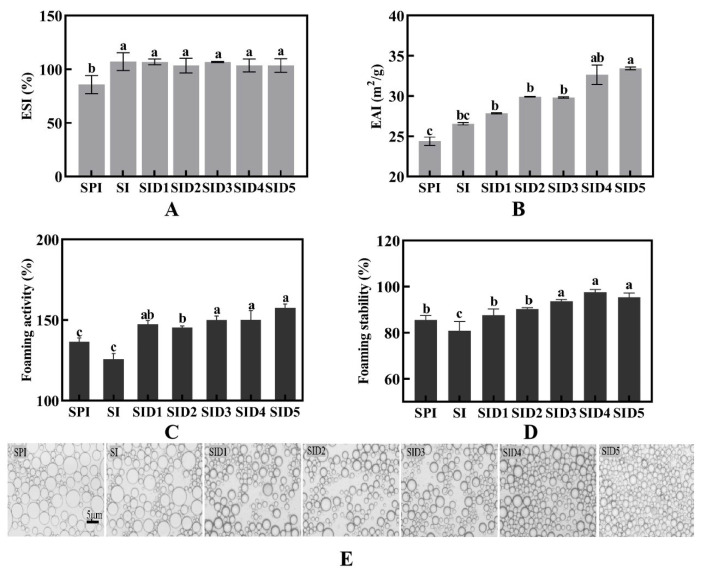
Emulsifiability (**A**,**B**), foamability (**C**,**D**), and emulsion micrograph (**E**) of SPI, SPI-INU (SI) binary complex, and SPI-INU-DMY (SID) ternary complexes at different DMY concentrations. Different lowercase letters (a–c) indicate significant differences (*p* < 0.05).

The emulsifying activity index (EAI) measures the capacity of proteins to rapidly adsorb onto both hydrophobic and hydrophilic phases, which is critical for the generation of stable emulsions. The emulsion stability index (ESI) reflects the emulsion’s resistance to gravitational settling [52]. Figure 7A,B illustrate the ESI and EAI for SPI and complexes. The ESI and EAI of SPI were 84.40% and 24.39 m^2^/g. When interacting with INU through a Maillard reaction, the ESI and EAI of SPI were increased to 107.24% and 27.56 m^2^/g, respectively. This is because when SPI reacts with INU, it creates a spatial site resistance that prevents the aggregation of the droplets and leads to SPI having higher solubility and easier adsorption at the oil–water interface. Moreover, INU can improve the viscosity of the emulsion, which acts as a fixation for the emulsion droplets and thus improves the stability of the emulsion. This is similar to the result that chitosan improves the emulsifying properties of SPI [56]. The addition of DMY did not have a significant impact on the ESI. As the concentration of DMY increased, the EAI steadily improved. This showed that soy protein isolate was better able to adsorb at the oil–water interface. This is because the DMY is amphiphilic, so it increases the hydrophobicity of the protein [20]. It might also be the result of more proteins unfolding and curving irregularly, which could increase the droplet interface’s capacity to bind water and oil [3].

The micromorphology of emulsions was observed using an optical microscope (Figure 7E). As the concentration of DMY increased, the emulsions’ droplets presented smaller sizes and a more uniform distribution. This proves that the ternary complexes’ emulsifying qualities were improved [41].

#### 3.3.3. Foaming Properties

Many foods are composed of bubbles dispersed in water. However, due to coalescence and Ostwald ripening, food foams collapse with time. Thus, foaming agents are needed to quickly absorb at the air–water interface and to generate small bubbles that resist coalescence, expansion, and contraction [26]. The molecular flexibility, surface activity, and hydrophobicity of proteins are all very important in the process of foam formation [62]. As shown in Figure 7C,D, the foaming activity and stability run in the following order: ternary complex (protein/polysaccharide/polyphenol) > protein > binary complex (protein/polysaccharide). This may be due to the addition of DMY, which improves molecular flexibility and allows more protein molecules to transfer to the air–water interface, thus improving the foaming properties [63]. A similar result was found for anthocyanins in the foaming properties of soybean isolate proteins [41]. When ferulic acid (FA) was added, the foaming activity and stability of ovalbumin were enhanced by 21% and 19%, respectively [63]. However, polyphenols (tannins) may reduce the foaming properties of proteins [64]. It has been shown that the fewer hydroxyl and ortho-aromatic amino acids a polyphenol contains, the more it improves the foaming properties of the protein [65]. Therefore, combining suitable polyphenols can improve the foaming characteristics of proteins.

### 3.4. Functional Properties of SPI-INU-DMY Complexes

#### 3.4.1. Antioxidant Capacity

**Figure 8:**
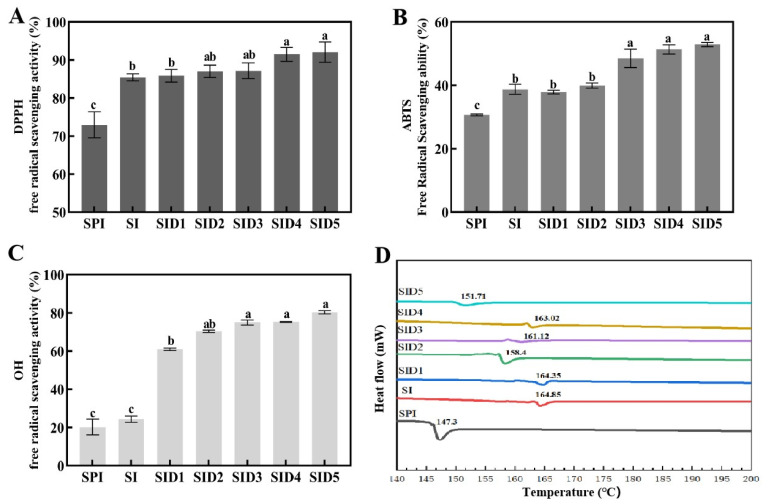
DPPH, ABTS, OH radical scavenging ability (**A**–**C**), and DSC (**D**) of SPI, SPI-INU (SI) binary complex, and SPI-INU-DMY (SID) ternary complexes at different DMY concentrations. Different lowercase letters (a–c) indicate significant differences (*p* < 0.05).

The external environment may influence emulsions, resulting in the aggregation of oil droplets and a decrease in stability. Solid particles with antioxidant properties can effectively capture free radicals and combine with oxidizing substances in the reduction reaction to slow the oxidation of oil droplets, which makes the emulsion more stable [66]. To evaluate the antioxidant activity of the complexes with differing DMY concentrations, the DPPH, ABTS, and OH free radical scavenging rates of the samples were evaluated. As can be seen in Figure 8A–C, INU and DMY significantly improved the antioxidant activity of SPI. The addition of INU resulted in an enhancement of the DPPH, ABTS cation, and OH free radical scavenging rates by 17.08%, 26.38%, and 20.66%, respectively. The DPPH radical scavenging ability of the SI increased. This is because inulin provides hydrogen bonding, thus forming stable DPPH-H atoms [8]. DMY has a high free radical scavenging capacity and can help SI form a more stable structure. After the addition of DMY, the antioxidant activity of the SID complex is enhanced. The higher the concentration of DMY added, the better its antioxidant capacity becomes. Similarly, the non-covalent modification of soy isolate protein with apple polyphenols increased the antioxidant capacity of the soy isolate proteins [7].

#### 3.4.2. Thermal Stability

The results of the DSC analysis of the different samples are shown in Figure 8D. All samples showed a single peak at 145–165 °C. SPI underwent a thermo-absorptive transition with a peak temperature of about 147.3 °C. The complexes increased the thermal transition temperature compared to the single protein. The SID ternary complex has a lower thermal denaturation temperature than the SI binary complex. The non-covalent connection between the SI complex and DMY further affects the electrostatic repulsion and hydrophobicity of the protein molecule, thus reducing thermal stability [61]. The reason why the thermal stability of SID is lower than that of SI may also be related to DMY changing the rigid structure of SI. A similar trend was also found in the incorporation of EGCG into pea proteins, where the denaturation temperature decreased with an increasing EGCG content, probably due to the change in the protein structure during the binding process with polyphenols [67].

## 4. Conclusions

In this study, dihydromyricetin (polyphenol) was added to an SPI/INU complex that was obtained through a Maillard reaction. DMY forms a more flexible and compact SID ternary complex with SI through hydrophobic interaction and hydrogen bonding, thus changing the structure of SI. The addition of DMY mainly unfolds the structure of SPI and changes its conformation, making the structure of SI more compact and finally obtaining a complex with a small particle size. However, high-concentration DMY (2.5 mg/mL) will cause the aggregation of SPI and SI, which is not conducive to the stability of the SID ternary complex. Generally, adding DMY (0.5 mg/mL~2.5 mg/mL) to the SI binary complex can improve the emulsifying property, foaming property, and antioxidant activity of the SI binary complex. Therefore, this study provides a reference for adding more polyphenols to the protein/polysaccharide system in more binding ways and expands the research and application of ternary complexes in fields such as food and emulsions. The constructed SID complexes have the potential to serve as antioxidant emulsifiers in emulsion-based foods, thereby expanding the use of SPI in the food industry and enhancing the use of INU and DMY in emulsion applications.

## Data Availability

The original contributions presented in this study are included in the article; further inquiries can be directed to the corresponding author.

## References

[1] Mo H.P., Li Q., Liang J.R., Ou J.J., Jin B. (2021). Investigation of physical stability of Pickering emulsion based on soy protein/β-glucan/coumarin ternary complexes under subcritical water condition. Int. J. Food Sci. Technol..

[2] Najari Z., Dokouhaki M., Juliano P., Adhikari B. (2024). Advances in the application of protein-polysaccharide-polyphenol ternary complexes for creating and stabilizing Pickering emulsions. Future Foods.

[3] Li H.J., Zhang J., Wu Y.F., Ren C., Qiu X.B., Li H.B., Sun G.L., Li K.W., Yu J.H. (2024). Preparation and characterization of whey protein isolate-dextran-proanthocyanidins ternary complexes: Formation mechanism, physicochemical stability. Food Hydrocoll..

[4] Mao L.K., Wang W.Y., Tai K.D., Yuan F., Gao Y.X. (2017). Development of a soy protein isolate-carrageenan-quercetagetin non-covalent complex for the stabilization of beta-carotene emulsions. Food Funct..

[5] Xu X.Y., Li L., Ma C.M., Li D., Yang Y., Bian X., Fan J., Zhang N., Zuo F. (2023). Soy protein isolate-citrus pectin-gallic acid ternary composite high internal phase Pickering emulsion for delivery of beta-carotene: Physicochemical, structural and digestive properties. Food Res. Int..

[6] Dai S.C., Liao P.L., Wang Y.L., Tian T., Tong X.H., Lyu B., Cheng L., Miao L.M., Qi W.J., Jiang L.Z. (2023). Soy protein isolate-catechin non-covalent and covalent complexes: Focus on structure, aggregation, stability and in vitro digestion characteristics. Food Hydrocoll..

[7] Song Z.C., Zhang H., Niu P.F., Shi L.S., Yang X.Y., Meng Y.H., Wang X.Y., Gong T., Guo Y.R. (2023). Fabrication of a novel antioxidant emulsifier through tuning the molecular interaction between soy protein isolates and young apple polyphenols. Food Chem..

[8] Jiang W., Wang Y.Y., Ma C.C., McClements D.J., Liu F.G., Liu X.B. (2022). Pea protein isolate-inulin conjugates prepared by pH-shift treatment and ultrasonic-enhanced glycosylation: Structural and functional properties. Food Chem..

[9] Parolia S., Maley J., Sammynaiken R., Green R., Nickerson M., Ghosh S. (2022). Structure—Functionality of lentil protein-polyphenol conjugates. Food Chem..

[10] Virk M.S., Virk M.A., Liang Q., Sun Y., Zhong M., Tufail T., Rashid A., Qayum A., Rehman A., Ekumah J.N. (2024). Enhancing storage and gastroprotective viability of *Lactiplantibacillus plantarum* encapsulated by sodium caseinate-inulin-soy protein isolates composites carried within carboxymethyl cellulose hydrogel. Food Res. Int..

[11] Li J.P., Yang J.J., Li J.Z., Gantumur M.A., Wei X., Oh K.C., Jiang Z.M. (2023). Structure and rheological properties of extruded whey protein isolate: Impact of inulin. Int. J. Biol. Macromol..

[12] López-Castejón M.L., Bengoechea C., Espinosa S., Carrera C. (2019). Characterization of prebiotic emulsions stabilized by inulin and β-lactoglobulin. Food Hydrocoll..

[13] Ma X.B., Yan T.Y., Hou F.R., Chen W.J., Miao S., Liu D.H. (2019). Formation of soy protein isolate (SPI)-citrus pectin (CP) electrostatic complexes under a high-intensity ultrasonic field: Linking the enhanced emulsifying properties to physicochemical and structural properties. Ultrason. Sonochem..

[14] Wang M.M., Yang S., Sun N., Zhu T.T., Lian Z.T., Dai S.C., Xu J., Tong X.H., Wang H., Jiang L.Z. (2024). Soybean isolate protein complexes with different concentrations of inulin by ultrasound treatment: Structural and functional properties. Ultrason. Sonochem..

[15] Nikbakht Nasrabadi M., Sedaghat Doost A., Mezzenga R. (2021). Modification approaches of plant-based proteins to improve their techno-functionality and use in food products. Food Hydrocoll..

[16] Jiang S.S., Ding J.Z., Andrade J., Rababah T.M., Almajwal A., Abulmeaty M.M., Feng H. (2017). Modifying the physicochemical properties of pea protein by pH-shifting and ultrasound combined treatments. Ultrason. Sonochem..

[17] Zhang Y., Di R.P., Zhang H.X., Zhang W.J., Wu Z.L., Liu W., Yang C.Y. (2020). Effective recovery of casein from its aqueous solution by ultrasonic treatment assisted foam fractionation: Inhibiting molecular aggregation. J. Food Eng..

[18] Tian T., Teng F., Zhang S., Qi B., Wu C., Zhou Y., Li L., Wang Z.J., Li Y. (2019). A Study of Structural Change during In Vitro Digestion of Heated Soy Protein Isolates. Foods.

[19] Geng S., Liu X.L., Ma H.J., Liu B.G., Liang G.Z. (2021). Multi-scale stabilization mechanism of pickering emulsion gels based on dihydromyricetin/high-amylose corn starch composite particles. Food Chem..

[20] Geng S., Jiang Z.J., Ma H.J., Pu P., Liu B.G., Liang G.Z. (2021). Fabrication and characterization of novel edible Pickering emulsion gels stabilized by dihydromyricetin. Food Chem..

[21] Liu X.L., Geng S., He C.Y., Sun J.L., Ma H.J., Liu B.G. (2021). Preparation and characterization of a dihydromyricetin-sugar beet pectin covalent polymer. Food Chem..

[22] Cao Y.J., Zang Z.X., Zhang L., Han G.X., Yu Q.L., Han L. (2023). Hydroxypropyl methyl cellulose/soybean protein isolate nanoparticles incorporated broccoli leaf polyphenol to effectively improve the stability of Pickering emulsions. Int. J. Biol. Macromol..

[23] Qin W.L., Tang S.T., Chen C.W., Xie J. (2024). Preparation and characterization of cinnamon essential oil Pickering emulsion stabilized by zein/carboxylated cellulose nanocrystals composite nanoparticles. Food Hydrocoll..

[24] Wang P.P., Wang W.D., Chen C., Fu X., Liu R. (2020). Effect of Fructus Mori. bioactive polysaccharide conjugation on improving functional and antioxidant activity of whey protein. Int. J. Biol. Macromol..

[25] Wu J.D., Shi M.X., Li W., Zhao L.H., Wang Z., Yan X.Z., Norde W., Li Y. (2015). Pickering emulsions stabilized by whey protein nanoparticles prepared by thermal cross-linking. Colloids Surf. B.

[26] Li C.H., Dai T.T., Chen J., Li X., Li T., Liu C.M., McClements D.J. (2021). Protein-polyphenol functional ingredients: The foaming properties of lactoferrin are enhanced by forming complexes with procyanidin. Food Chem..

[27] Jing Y.J., Huang J.H., Yu X.Q. (2018). Preparation, characterization, and functional evaluation of proanthocyanidin-chitosan conjugate. Carbohydr. Polym..

[28] Li D., Zhao Y., Wang X., Tang H.L., Wu N., Wu F., Yu D.Y., Elfalleh W. (2020). Effects of (+)-catechin on a rice bran protein oil-in-water emulsion: Droplet size, zeta-potential, emulsifying properties, and rheological behavior. Food Hydrocoll..

[29] Yang W., Xu C.Q., Liu F.G., Sun C.X., Yuan F., Gao Y.X. (2015). Fabrication Mechanism and Structural Characteristics of the Ternary Aggregates by Lactoferrin, Pectin, and (−)-Epigallocatechin Gallate Using Multispectroscopic Methods. J. Agric. Food Chem..

[30] Dai T.T., Li T., Li R.Y., Zhou H.L., Liu C.M., Chen J., McClements D.J. (2020). Utilization of plant-based protein-polyphenol complexes to form and stabilize emulsions: Pea proteins and grape seed proanthocyanidins. Food Chem..

[31] Sun Y.J., Li C.Y., Li W., Li G.K., Zhang T., Miao M. (2024). Impact of particle size on the functionality of corn-derived dietary fiber-phenolic acid complexes. Int. J. Biol. Macromol..

[32] Liu Z.Y., Zheng K.W., Yan R.Z., Tang H.H., Jia Z.Y., Zhang Z.Q., Yang C., Wang J.M. (2024). Effects of different solid particle sizes on oat protein isolate and pectin particle-stabilized Pickering emulsions and their use as delivery systems. Food Chem..

[33] Xu J.J., Ji F.Y., Luo S.Z., Jiang S.T., Yu Z.Y., Ye A.Q., Zheng Z. (2024). Fabrication of soy protein–polyphenol covalent complex nanoparticles with improved wettability to stabilize high-oil-phase curcumin emulsions. J. Sci. Food Agric..

[34] Peng K.D., Li Y., Sun Y., Xu W., Wang H.X., Zhang R., Yi Y. (2023). Lotus Root Polysaccharide-Phenol Complexes: Interaction, Structure, Antioxidant, and Anti-Inflammatory Activities. Foods.

[35] Feng T.T., Wang X.J., Wang X.W., Zhang X.M., Gu Y., Xia S.Q., Huang Q.R. (2021). High internal phase pickering emulsions stabilized by pea protein isolate-high methoxyl pectin-EGCG complex: Interfacial properties and microstructure. Food Chem..

[36] Zhang L.J., Zhou C.F., Xing S.H., Chen Y.N., Su W.T., Wang H.T., Tan M.Q. (2023). Sea bass protein-polyphenol complex stabilized high internal phase of algal oil Pickering emulsions to stabilize astaxanthin for 3D food printing. Food Chem..

[37] Ma L.L., Yang X.F., Huo J.Y., Li S.G. (2025). Study on the mechanism of polyphenols regulating the stability of pea isolate protein formed Pickering emulsion based on interfacial effects. Food Chem..

[38] He W.S., Wang Q.Z., Li Z.S., Li J., Zhao L.Y., Li J.J., Tan C., Gong F.Y. (2023). Enhancing the Stability and Bioaccessibility of Tree Peony Seed Oil Using Layer-by-Layer Self-Assembling Bilayer Emulsions. Antioxidants.

[39] Yang J., Mao L., Yang W., Sun C.X., Dai L., Gao Y.X. (2018). Evaluation of non-covalent ternary aggregates of lactoferrin, high methylated pectin, EGCG in stabilizing beta-carotene emulsions. Food Chem..

[40] Chen Y., Yao M.Y., Peng S., Fang Y.J., Wan L.T., Shang W.T., Xiang D., Zhang W.M. (2023). Development of protein-polyphenol particles to stabilize high internal phase Pickering emulsions by polyphenols’ structure. Food Chem..

[41] Sui X.N., Sun H.B., Qi B.K., Zhang M., Li Y., Jiang L.Z. (2018). Functional and conformational changes to soy proteins accompanying anthocyanins: Focus on covalent and non-covalent interactions. Food Chem..

[42] Dai T.T., McClements D.J., Hu T., Chen J., He X.M., Liu C.M., Sheng J.F., Sun J. (2022). Improving foam performance using colloidal protein-polyphenol complexes: Lactoferrin and tannic acid. Food Chem..

[43] Li W.H., Han S., Huang H.C., McClements D.J., Chen S., Ma C.C., Liu X.B., Liu F.G. (2024). Fabrication, characterization, and application of pea protein isolate-polyphenol-iron complexes with antioxidant and antibacterial activity. Food Hydrocoll..

[44] Song Y.q., Yao G.L., Chen J., Li N. (2023). Effect of β-cyclodextrin on whey protein-epigallocatechin gallate interaction. Ind. Crops Prod..

[45] Liu J., Zhang Y.M., Liu J.Y., Zhang H.J., Gong L.X., Li Z.F., Liu H.Z., Wang Z.Y. (2024). Effect of non-covalently bound polyphenols on the structural and functional properties of wheat germ protein. Food Hydrocoll..

[46] Pi X.W., Fu G.M., Dong B., Yang Y.L., Wan Y., Xie M.Y. (2021). Effects of fermentation with Bacillus natto on the allergenicity of peanut. LWT.

[47] Sun J., Liu T.M., Zhang F., Huang Y.Q., Zhang Y., Xu B. (2022). Tea polyphenols on emulsifying and antioxidant properties of egg white protein at acidic and neutral pH conditions. LWT.

[48] Puppo M.C., Speroni F., Chapleau N., de Lamballerie M., Añón M.C., Anton M. (2005). Effect of high-pressure treatment on emulsifying properties of soybean proteins. Food Hydrocoll..

[49] Li H., Chen Z.Z., Qian Y., Dai Y., Ping Y.L., Wang Q.Y., Fang X.X., Liu X.R., Zhao B.B. (2024). Effect of tea polyphenols on the formation of advanced glycation end products (AGEs), functional and structural properties of modified protein in Maillard reaction. LWT.

[50] Yang J., Zhao Y.J., Shan B.S., Duan Y.Q., Zhou J., Cai M.H., Zhang H.H. (2023). Study on the interaction and functional properties of Dolichos lablab L. protein-tea polyphenols complexes. Int. J. Biol. Macromol..

[51] Liu F.G., Sun C.X., Wang D., Yuan F., Gao Y.X. (2015). Glycosylation improves the functional characteristics of chlorogenic acid–lactoferrin conjugate. RSC Adv..

[52] Chen H.H., Jiang H., Chen Y., Qu Y.P., Wang Y.S. (2022). A pH-controlled curcumin-loaded emulsion stabilized by pea protein isolate-maltodextrin-epigallocatechin-3-gallate: Physicochemical properties and in vitro release properties. Colloids Surf. A.

[53] Ge G., Guo W.X., Zheng J.B., Zhao M.M., Sun W.Z. (2021). Effect of interaction between tea polyphenols with soymilk protein on inactivation of soybean trypsin inhibitor. Food Hydrocoll..

[54] Wang T., Chen X., Wang W.N., Wang L.Q., Jiang L.Z., Yu D.Y., Xie F.Y. (2021). Effect of ultrasound on the properties of rice bran protein and its chlorogenic acid complex. Ultrason. Sonochem..

[55] Tian R., Han X.E., Tian B., Li G.L., Sun L.A., Tian S.F., Qin L.X., Wang S. (2023). Effects of covalent binding of different polyphenols on structure, rheology and functional properties of whey protein isolate. LWT.

[56] Xu Z.Z., Huang G.Q., Xu T.C., Liu L.N., Xiao J.X. (2019). Comparative study on the Maillard reaction of chitosan oligosaccharide and glucose with soybean protein isolate. Int. J. Biol. Macromol..

[57] Liu X.J., Song Q.B., Li X., Chen Y.X.j, Liu C., Zhu X., Liu J., Granato D., Wang Y.J., Huang J.B. (2021). Effects of different dietary polyphenols on conformational changes and functional properties of protein–polyphenol covalent complexes. Food Chem..

[58] Chen Y.L., Chen Y., Jiang L.Z., Huang Z.X., Zhang W.M., Yun Y.H. (2023). Improvement of emulsifying stability of coconut globulin by noncovalent interactions with coffee polyphenols. Food Chem. X.

[59] Wang H.P., Li Z.H., Meng Y., Lv G.H., Wang J.P., Zhang D., Shi J.Y., Zhai X.D., Meng X.R., Zou X.B. (2024). Co-delivery mechanism of curcumin/catechin complex by modified soy protein isolate: Emphasizing structure, functionality, and intermolecular interaction. Food Hydrocoll..

[60] Zhao Y., Wang X., Li D., Tang H.L., Yu D.Y., Wang L.Q., Jiang L.Z. (2020). Effect of anionic polysaccharides on conformational changes and antioxidant properties of protein-polyphenol binary covalently-linked complexes. Process Biochem..

[61] Ke C.X., Li L. (2024). Modification mechanism of soybean protein isolate-soluble soy polysaccharide complex by EGCG through covalent and non-covalent interaction: Structural, interfacial, and functional properties. Food Chem..

[62] Dombrowski J., Gschwendtner M., Kulozik U. (2017). Evaluation of structural characteristics determining surface and foaming properties of β-lactoglobulin aggregates. Colloids Surf. A.

[63] Chang K.F., Liu J.B., Jiang W., Zhang R.X., Zhang T., Liu B.Q. (2020). Ferulic acid-ovalbumin protein nanoparticles: Structure and foaming behavior. Food Res. Int..

[64] Zhan F.C., Li J., Wang Y.T., Shi M.Q., Li B., Sheng F. (2018). Bulk, Foam, and Interfacial Properties of Tannic Acid/Sodium Caseinate Nanocomplexes. J. Agric. Food Chem..

[65] Wen H.D., Zhang D.J., Ning Z.Z., Li Z., Zhang Y., Liu J.B., Zhang T. (2023). How do the hydroxyl group number and position of polyphenols affect the foaming properties of ovalbumin?. Food Hydrocoll..

[66] Di X.H., Li Y.C., Qin X.G., Wang Q., Liu G. (2024). Investigating the effect of whey protein isolate:proanthocyanidin complex ratio on the stability and antioxidant capacity of Pickering emulsions. Int. J. Biol. Macromol..

[67] Han S., Cui F.Z., McClements D.J., Xu X.F., Ma C.C., Wang Y.T., Liu X.B., Liu F.G. (2022). Structural Characterization and Evaluation of Interfacial Properties of Pea Protein Isolate-EGCG Molecular Complexes. Foods.

